# Cancer Risk in Type 2 Diabetes Mellitus: Metabolic Links and Therapeutic Considerations

**DOI:** 10.1155/2011/708183

**Published:** 2011-06-01

**Authors:** Grace Sun, Sangeeta R. Kashyap

**Affiliations:** Department of Endocrinology, Diabetes, and Metabolism, Cleveland Clinic, Cleveland, OH 44195, USA

## Abstract

Type 2 diabetes mellitus (DM2) is increasing in incidence, creating worldwide public health concerns and impacting morbidity and mortality rates. An increasing number of studies have demonstrated shared associations between DM2 and malignancy, including key clinical, biochemical, and metabolic commonalities. This paper will attempt to explore the relationship between the various types of cancer and diabetes, the common metabolic pathways underlying cancer development, and the potential impact of various antidiabetes therapies on cancer risk.

## 1. Introduction

The association between diabetes and cancer was described long before the 21st century, as far back as 1885 [1]. DM2 continues to increase in prevalence affecting 17.9 million Americans with 5.7 million undiagnosed, costing an estimated 174 billion healthcare dollars [2]. Cancer, second only to heart disease in mortality, is also on the rise with estimated costs in the US of $263.8 billion [3]. These two high-impact diseases share factors which influence their development and progression, important in modifying each other's outcome. 

### 1.1. Clinical Factors Underlying Cancer Risk

**Figure 1:**
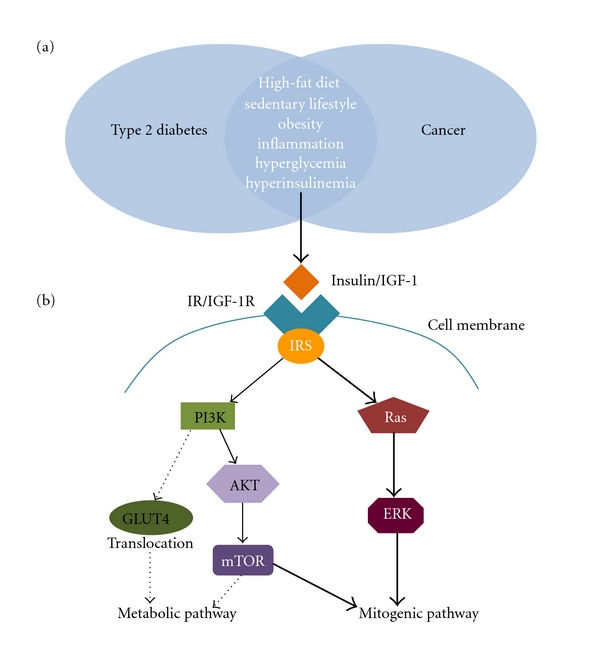
The “big picture” between DM2 and cancer. (a) The shared metabolic factors underlying both DM2 and cancer, including visceral adiposity, inflammation, hyperglycemia, and hyperinsulinemia lead to (b) increased insulin receptor substrate (IRS) stimulating the phosphorylation of Ras signaling proteins and potentially increasing tumor cell growth and proliferation. IRS-associated PI3K signaling is compromised by insulin resistant states, such as in DM2, and downstream GLUT4 translocation is disrupted. This disruption drives PI3K signaling towards AKT/mTOR. AKT and mTOR can affect both the metabolic and mitogenic pathway, but because of the signaling dysfunction, AKT and mTOR are driven towards the mitogenic pathway.

Common nonmodifiable (age, sex, and race/ethnicity) and modifiable (weight, diet, physical activity, tobacco use, and alcohol use) risk factors underlie the development of cancer and DM2. Figure 1(a) illustrates the general risk factors for cancer development in diabetes. It is known that both DM2 and cancer increase with age; those ≥55 years comprise almost 80% of newly diagnosed cancers and 23.1% of patients ≥ 60 years develop DM2 compared to 10.7% of younger adults [4, 5]. Men have slightly more cancer and diabetes compared to women after adjusting for other risks [4–6]. African-Americans as a subgroup appear hardest hit with regards to cancer-related deaths, DM2, and obesity compared to non-Hispanic white counterparts [4, 5]. Central obesity, a marker of insulin resistance and a key player in both DM2 and the metabolic syndrome, has also been linked to breast, colorectal, liver, and endometrial, malignancies [5]. Dietary choices high in glycemic load and saturated fat and low in fiber accompanied by reduced physical activity also increase the risk for DM2 and malignancy, particularly for the colon, endometrium and breast [5, 7]. Moreover, studies show that tobacco and excess alcohol usage linked to cancer can also worsen diabetes complications [5]. 

### 1.2. Types of Cancers Linked to DM2 and Common Metabolic Pathways

Diabetes has been recognized as a key factor contributing to the development of solid organ malignancies including liver, pancreas, colorectal, breast, endometrial, uterine, and bladder [3, 5, 8–18]. The two cancers showing the strongest association to DM2 are those of the liver [1, 5, 9, 18–20] and pancreas [1, 5, 18–20]. Not only does there appear to be an association between DM2 and cancer, but having both diagnoses may increase mortality, regardless of the type of cancer [9, 21]. Studies performed in Japan, Korea, and Hong Kong demonstrated increased cancer risk [19, 20, 22] and mortality [19, 22] among participants with DM2, especially those with poorly controlled DM2 based on HbA1c [22] and fasting serum glucose levels [19]. A recent meta-analysis of 23 articles indicated a 41% increase in cancer mortality related to endometrial, breast, and colorectal cancer in patients with preexisting diabetes as compared to normoglycemic individuals [21]. In the American Cancer Society Cancer Prevention Study II [9] that enrolled over 1 million patients, adults with diabetes and cancer had greater mortality. In contrast, increased mortality was not found in subjects with diabetes in the National Health and Nutrition Examination Survey Mortality Followup [23]. 

Considering the associations between diabetes and various malignancies, can mechanisms related to diabetes development predispose to oncogenic expression? There appears to be a critical interplay between hyperglycemia, hyperinsulinemia, and adiposity, particularly central adiposity, creating a low-grade chronic inflammatory state. These elements are proposed to connect cancer development and progression to DM2 which may also influence the response to anticancer therapy. Since insulin, glycemia, adiposity, and inflammation can be modified by antidiabetes pharmacotherapy, a better understanding of their pathophysiologic links to cancer may allow us to more effectively target DM2 treatment. 

Insulin, because of its known mitogenic effects, has been implicated as a key mediator in the complex mechanisms involved in carcinogenesis. Both insulin and insulin-like growth factor-1 (IGF-1) have affinity to both the insulin receptor (IR) and IGF-1 receptor (IGF-1R) because of similar structural homology. But it is important to recognize that insulin's affinity for the IR is upwards of 1000-fold greater than for IGF-1R [24]. Both IGF-1 and IGF-1R tend to have stronger mitogenic and antiapoptotic effects, and the hyperinsulinemia that occurs in insulin-resistant individuals may enhance this effect [1, 5, 18, 24]. Insulin may also indirectly promote cancer development via IGF-1. Insulin decreases IGF-binding protein-1, and possibly IGF-binding protein 2, which may increase the bioactive form of the growth factor, IGF-1 [18, 24] In addition, malignant cells predominantly express the A isoform of the IR (IR-A) or hybrid IR-A/IGF-1R forms, which have more mitogenic effects than the B isoform IR [5, 18, 25]. Thus, in the hyperinsulinemic individual, insulin's mitogenic properties may further oncogenic proliferation. 

Furthermore, hyperinsulinemia may ultimately upregulate the insulin mitogenic pathway compared to the insulin metabolic pathway. Insulin resistant states such as obesity, metabolic syndrome, and DM2 cause impairment of downstream GLUT4 translocation by disruption of insulin receptor substrate-1 (IRS-1) associated PI3K signaling in the metabolic pathway of insulin [26]. Insulin, to a lesser extent than IGF-1, stimulates cellular growth and protein synthesis through the protein kinase B (PKB) system and activation of mTOR [27]. Abnormal IRS-1 phosphorylation from overactivation of mTOR creates a negative feedback loop that attenuates the metabolic pathway in hyperinsulinemia [18, 27]. IRS-2 expression by insulin phosphorylation leads to increased ERK activation, a mitogen-activated protein kinase pathway (MAPK), because the mitogen pathways mediated by mTOR and Ras remain intact [18, 26, 27]. This drive towards the mitogen pathway with hyperinsulinemia leads to enhanced cell growth and survival. Figure 1(b) illustrates the potential molecular pathways, mediated by insulin, which lead to cancer. Moreover, hyperglycemia may not only promote tumorigenesis indirectly by stimulating insulin production, but also may have a direct effect, since cancer cells depend on glycolysis for energy [6, 17].

Central adiposity highly correlates with insulin resistance and is a key player in metabolic derangements associated with diabetes. Adipose tissue-derived cytokines, free fatty acids, and other vascular factors released from the visceral compartment have been shown to induce insulin resistance, lipid abnormalities, glucose intolerance, and low grade inflammation that collectively creates an environment ripe for cancer development and progression. In contrast, adiponectin is of particular interest for cancer prevention because of its unique metabolic properties which include the ability to decrease hepatic gluconeogenesis, to increase insulin sensitivity, and to reduce adipogenic inflammation [28–31]. Working in opposition to adiponectin are resistin, visfatin, TNF *α*, IL-6, and MCP-1. These factors may increase cancer risk by perpetuating insulin resistance, hyperglycemia and inflammation [25, 28–34]. The 30% mortality reduction related primarily to cancer and cardiovascular events following massive weight loss after bariatric surgery in morbidly obese subjects enrolled in the Swedish obesity study cohort further supports the contribution of adipocytokines in carcinogenesis [35]. However, the precise mechanisms related to lower cancer risk following surgical weight loss remains unclear.

Breast cancer is one of the more widely studied malignancies with respect to its connection to diabetes. Some studies showed an increased risk of breast malignancy in individuals with diabetes [8–11, 14–16, 18], whereas others did not [36–39]. Although most studies were retrospective case-control or cohort studies [8–10, 14–18, 36–38], one of two large-scale prospective studies of more than 97,000 Japanese women showed no increase in breast cancer risk [20], whereas another with more than 116,000 women enrolled in the Nurses' Health Study, showed a mild, but significantly increased, cancer risk with a hazard ratio of 1.17 (95% CI of 1.01–1.35) in those with diabetes [11]. Additionally, many studies demonstrate greater cancer risk in postmenopausal women with DM2 than their younger, premenopausal counterparts [10, 14, 21]. Elevations in plasma estrogen levels have been associated with postmenopausal breast cancer risk in the nondiabetes population [1, 34]. In insulin-resistant postmenopausal women, elevated serum insulin levels reduce sex-hormone-binding globulin (SHBG), resulting in increased estrogen bioavailability possibly explaining this increased risk [1, 5, 34, 40]. The elevation of free estrogens is also seen in postmenopausal DM2 even after adjusting for the degree of obesity [5, 34]. Furthermore, insulin has been shown to be mitogenic in human mammary epithelial cells, but was less mitogenic compared to IGF-1 [24]. In obesity, IGF-1 is an important link to breast cancer [34]. Hyperinsulinemia could stimulate IGF-1R and hybrid receptors although the clinical significance of this in breast cancer development is still unclear. It is important to remember that IGF-1 is much more mitogenic than insulin, and an insulin concentration 100 times that of IGF-1 was needed to elicit equivalent breast cancer cell growth [24]. *In vitro *animal models showed IGF-1 to be a significant risk factor in mammary tumor onset and development [41]. 

A significant risk for colorectal cancer in DM2 patients as compared to those without diabetes has been reported in a number of retrospective and prospective studies examining this relationship. Some studies show an increased risk only in men, whereas others document a risk in both genders [18, 42–47]. As with breast cancer, the underlying factors appear to be elevated glucose, insulin and IGF-1 levels. For example, *in vitro* systems point to enhanced colonic tumor cell growth with elevations of both insulin and IGF-1 levels [48–51]. Decreased IGF binding proteins may also play a role in colorectal cancer development as suggested by one [51] but not other studies [50, 52, 53].

Because the liver and pancreas are two main target organs for insulin metabolism, it is not surprising that DM2 and hepatocellular carcinoma share a link. The incidence of hepatocellular carcinoma has been reported to be higher in those with DM2 in both sexes, with a greater risk in men [1] and in those with concomitant hepatitis C infection [18, 54–61]. Some studies show that DM2 increases hepatocellular cancer only in the presence of hepatitis or cirrhosis [56, 57, 59, 60], whereas others observe that the association exists in the absence of those characteristics [18, 54, 58, 61].

Other cancers linked to diabetes and its metabolic features include endometrial, pancreatic, kidney, and possibly gastric malignancies [5, 42, 54, 62]. Older studies published in the early 1990s have indicated that individuals with diabetes ≥5 years appear to have an increased risk of pancreatic cancer [63, 64]. More recent studies show that pancreatic cancer risk occurs within the first decade of the diagnosis of DM2, and some argue that the highest risk is within 2-3 years of DM2 onset [65, 66]. Controversy regarding pancreatic cancer has evolved from the “chicken or egg” issue: does pancreatic cancer cause diabetes or does this malignancy result from diabetes? Some investigators would argue that diabetes is a result of pre-existing pancreatic cancer causing destruction of ß-cells via tumor infiltration [65]. The tumor burden would have to be extensive for a physical loss of islet cells to cause DM2. Newer hypotheses suggests that DM2 results more from peripheral insulin resistance and disruption of amylin secretion in *β*-cells [67, 68] from a paraneoplastic effect of the tumor cells, rather than a physical destruction of *β*-cells. One epidemiologic case-control study by Chari et al. suggest that pancreatic cancer may cause DM2 through alteration of glucose metabolism in the liver and skeletal muscle, thereby inducing glucose intolerance [66]. Moreover, elevated glucose levels are associated with increased pancreatic cancer mortality [63]. Regardless of the exact nature of the association, it is clear that a relationship exists between DM2 and pancreatic cancer. 

Interestingly, the presence of *Helicobacter pylori* may increase gastric cancer risk [69–71] in diabetes, possibly mediated through an insulin resistance/hyperinsulinemia pathway [70]. Increase in gastrin levels, decrease in somatostatin levels, and increase in neutrophilic and monocytic infiltration of the gastric mucosa in patients with *H. pylori* infection may lead to insulin resistance [70]. Eradication of *H. pylori* may mitigate the gastric cancer risk [72]. However, the prevalence findings of *H. pylori* in DM2 patients has been variable, and we do not yet clearly understand the clinical implications in this population. 

There are also studies arguing against an association between diabetes and cancer, specifically for ovarian, lung, and prostate malignancies [5, 42, 73, 74]. In prostate cancer, DM2 appears to have a protective effect, and like pancreatic cancer, there is a temporal relationship, with the protective effect occurring later in DM2 [62, 74]. Circulating insulin levels are inversely correlated with dihydrotestosterone, testosterone, and SHBG. Thus, it has been proposed that the lower testosterone levels that occur in DM2 may be a mechanism of decreased prostate cancer risk [74]. However, elevated circulating testosterone levels are not a consistent finding associated with prostate cancer [5, 9, 62]. Another suggested mechanism that may explain the time-associated inverse relationship is the change in insulin levels as DM2 progresses. IGF-1 levels have been associated with increased prostate cancer risk. Insulin downregulates IGF binding protein 1, and as insulin levels decrease over time through reduction in *β*-cell mass with DM2 disease progression, there is less downregulation of IGF-binding protein 1. Since IGF binding protein 1 controls the amount of IGF-1 free levels, less circulating insulin may indirectly mitigate prostate cancer risk [9, 62, 74, 75].

 The studies examining the relationship between DM2 and hematopoietic malignancies appear to show a positive relationship between diabetes and hematopoietic cancers [6, 37], but this finding is not consistent in all studies [42, 76]. Major limitations of these studies include self-reported data regarding the diagnosis of diabetes, which makes it difficult to clearly ascertain the exact links between diabetes and hematopoietic cancers. Oxidative stress and cellular damage related to hyperglycemia may be involved in the association between the two [22, 77, 78]. One prospective cohort study found a positive association between postload plasma glucose levels in non-diabetes patients and non-Hodgkin's lymphoma in men and a 3-fold increased mortality risk from multiple myeloma in women at the highest level of post-load plasma glucose levels [78]. Clearly, further studies are needed in this arena. 

### 1.3. Antidiabetes Therapy and Cancer Risk

#### 1.3.1. Insulin Analogues and Cancer

Insulin is a known mitogen that stimulates the MAPK pathway leading to increases in growth factors. In addition, insulin down-regulates IGF binding proteins which may contribute to sex-steroid dependent cancers in diabetes, particularly postmenopausal breast and endometrial malignancies. *In vitro *studies have shown that high insulin levels can affect angiogenesis and potentially propagate tumor progression by stimulating the mitogen pathway via both insulin and IGF receptors [92, 93]. A number of studies have examined the effect of exogenous insulin usage with cancer. These studies are summarized in Tables 1(b) and 1(c). An initial concern was raised by the provocative report of a retrospective German study suggesting an increased cancer risk with glargine use [87]. Since then, most large-scale studies have focused on glargine although other insulin analogues have been studied as well. The results from retrospective studies are conflicting, with some studies showing an increased risk of malignancy [22, 82, 85–87, 94], whereas others [82, 83, 88, 88, 89, 89, 90, 90, 91, 91] did not. 

*In vitro* studies may help explain the basis of the concern regarding insulin analogues, particularly glargine. Weinstein et al. studied the comparative mitogenic effect at supraphysiologic doses of regular insulin and insulin analogues glargine, detemir, lispro, and aspart to IGF-1 in cancer-derived cell lines from colon (HCT116), prostate (PC3), and breast (MCF-7) [95]. The insulin analogues glargine, detemir and lispro stimulated proliferation with a dose-dependent effect of all three cell lines but less than IGF-1. Regular and aspart insulin had the lowest mitogenic effect compared to the other insulin analogues and IGF-1 [95]. Furthermore, studies [95–97] indicate that glargine induced MCF-7 breast cancer cell proliferation to a significant level compared to the MCF-10 cells, but the stimulation was less compared to IGF-1. This discrepancy in responses between the two breast cancer cell lines is attributed to the differences in relative cellular IGF-1R and IR expression [24]. However, one study by Liefvendahl et al. found that glargine had little to no increased mitogenic effect in malignant cell lines for breast cancer (MCF-7 and SKBR-3) or osteosarcoma (SaOS-2) compared to human insulin [93], The caveat in this controversy may relate to a dose-time exposure to insulin treatment, particularly with glargine; that is, cumulative insulin intake over time may potentially increase the cancer risk and mortality [98]. Because of all the controversy, the FDA has concluded that glargine is safe, but it is awaiting longer-term prospective studies regarding malignancy potential.

### 1.4. Oral Hypoglycemic Agents and Cancer

Agents which treat or prevent DM2 might be expected to influence risk of cancer favorably. Current oral agents that treated DM2 fall into two broad general categories: insulin providing (secretagogues, metiglinides, and incretin analogs) and insulin-sensitizing (metformin and thiazolidinediones). The insulin sensitizers, metformin, and thiazolidinediones (TZDs) are promising cancer therapies, because they not only lower glucose, insulin and fatty acid levels, but also may have unique anticarcinogenic properties that will be discussed below. 

#### 1.4.1. Insulin Sensitizers (Biguanides and Thiazolidinediones)

Metformin is generally used as a first-line agent for DM2 treatment; it decreases hepatic glucose output and increases glucose disposal in muscle, thereby reducing levels of circulating serum glucose and insulin levels. Epidemiological studies in diabetic patients have shown that metformin may be neutral [99] or protective of cancer risk [64, 80, 81, 90, 100, 101].  Table 1(a) displays data from several population studies examining oral anti-diabetes agents and cancer risk. A meta-analysis of diabetes studies with 4,042 cancer events and 529 cancer deaths demonstrated a 31% reduction in relative risk with metformin versus other antidiabetic drugs [102]. *In vitro *cell culture studies as well as animal studies have clearly demonstrated antitumor properties of metformin [103–108]. Mice mammary tumor growth was reduced by metformin, indicating a potential chemotherapeutic role in human breast cancer therapy [103]. The mechanisms for the anti-tumor effects of metformin include an inhibition of cell proliferation, decrease cancer proliferation, with partial cell-cycle arrest in oncogenic cell lines with the activation of 5′ adenosine monophosphate-activated protein (AMP) and AMP-kinase (AMPK) [5]. AMPK is an essential mediator of the tumor suppressor LKB1. Because of its properties, AMPK could be used to suppress cancer cells containing loss-of-function LKB1 mutations, active B-Raf mutations, or in cancers associated with metabolic syndrome. The activation of AMPK reprograms cellular metabolism by acting on mTORC1, p53, fatty acid synthase, and other molecules involved with regulating cell growth and metabolism [106].

Thiazolidinediones, or TZDs as they are more commonly known (i.e., rosiglitazone and pioglitazone), bind to peroxisome proliferator-activated receptor (PPAR) gamma (*γ*) receptor molecules inside the cell nucleus which, when activated, result in transcription of a variety of genes. PPAR *γ* is an adipocyte transcription factor, stimulating differentiation of adipocytes as well as inhibiting inflammatory cytokine production [109]. This class of drugs reduces insulin levels by enhancing insulin action. PPAR *γ* activation results in reduced free fatty acids and eicosanoids and inhibition of VEGF-induced angiogenesis, amongst other actions [110]. Like metformin, TZDs inhibit cancer cell growth, potentiation, and proliferation, inducing apoptosis, at the *in vitro *level [5, 34]. Conversely, there are studies in rodents showing increased tumorigenesis by PPAR *γ* agonists. Epidemiologic studies have shown inconclusive results with a recent meta-analysis [111] showing no effect on malignancy risk. Overall, studies examining TZDs are limited because of the short-term exposure and limited cases of cancer at specific sites. A phase 1 trial using thiazolidinediones in combination with chemotherapy for refractory cancers was negative, but large-scale controlled studies are warranted [5, 112].

#### 1.4.2. Secretagogues (Sulfonylureas and Meglitinides)

Secretagogues stimulate insulin production by binding to specific cell receptors that result in depolarization of pancreatic beta cells [81]. Sulfonylurea (SFU) use, like insulin, has been implicated in cancer risk. Glitinides are less commonly used, and their association with cancer is unknown. A few observational studies have associated SFU use with higher cancer risk. However, the limitations of these studies relate to the lack of power given the small numbers of cancer events [80]. In addition, most studies have not addressed differences between the various SFU agents with one small study suggesting variable effects [81]. The effect of oral diabetic agents on incidence of malignancy was extracted from two randomized controlled trials, ADOPT (A Diabetes Outcome Progression Trial) and RECORD (Rosiglitazone Evaluated for Cardiovascular Outcomes and Regulation of Glycaemia in Diabetes). Despite a total study exposure of 39,000 person/years over a 4- to 6-year duration, neither trial demonstrated an advantage of metformin over rosiglitzone or SFUs on cancer rates. The hazard ratio for metformin versus rosiglitazone was close to 1.0 in both studies [99]. However, the data regarding SFUs is less clear with a nonstatistically significant reduction of 22% (95% CI 0.53–1.14) in ADOPT for metformin versus SFUs and 25% (95% CI 0.85–1.18) in RECORD for rosiglitazone versus SFUs [99].

#### 1.4.3. Alpha Glucosidase Inhibitors (Acarbose)

Acarbose, an *α*-glucosidase inhibitor, curbs postprandial blood glucose excursions by inhibiting the enzymatic degradation of carbohydrates in the small intestines brush border. Although cancer risk information regarding *α*-glucosidase inhibitors is limited, one study suggests that acarbose may augment butyrate, a colonocyte energy source with anticolon cancer effects. This particular study showed promising results of acarbose use to enhance fecal butyrate concentrations, which may favorably affect colonic neoplasia [113]. 

#### 1.4.4. DPP-IV Inhibitors and GLP-1 Agonists

Dipeptidyl peptidase-IV (DPP-IV) inhibitors, as well as glucagon-like peptide-1 (GLP-1) agonists, are relatively new medications in the arsenal of treatment of DM2. Sitagliptin, one of two available DPP-IV inhibitors in the US, initially made its debut in 2006 followed by the release of saxagliptin in 2009. The noninsulin injectable GLP-1 agonists, exenatide and liraglutide, were first introduced in the US in 2005, with a once-weekly option on the horizon. These therapies are effective in preserving ß-cell mass by inhibiting apoptosis as well as improving islet cell function [114, 115]. GLP-1 is an incretin hormone that stimulates up to 70% of glucose-dependent insulin secretion after an oral stimulus, and in non-DM2 patients, there is a quick first-phase insulin surge followed by a longer second-phase response. DM2 patients have approximately 80% decreased ß-cell function as a result of increased cell death from the oxidative stress of persistent hyperglycemia [114]. Subsequently, the first phase of insulin is lost in DM2. GLP-1 receptor agonists and the DPP-IV inhibitors, which inhibit the enzymatic breakdown of endogenous GLP-1, can restore ß-cell sensitivity to glucose, increase ß-cell mass, and improve overall islet cell function [114, 115].

Their relatively short-term use clinically does not permit meaningful data on malignancy potential or risks. Thus, only *in vitro* and animal studies may be used to address a potential risk. DPP-IV has a complex role in relationship to cancer and may influence all stages of cancer from apoptosis, migration, invasion, and metastasis to even chemotherapy sensitivity [116]. Sitagliptin showed increased pancreatic ductal hyperplasia in a small rodent model study [5]. Increased ductal cell turnover and ductal metaplasia may predispose to pancreatic cancer risk although one short-term study involving human pancreatic cancer cell lines did not result in cell proliferation with activation of GLP-1R signaling. This lack of cell proliferation even after activation of functional GLP-1R may be a result of GLP-1R expression in the transduction pathway activation in pancreatic cancer cells [117]. Of the two GLP-1 agonists, liraglutide was associated with thyroid C-cell hyperplasia/tumor growth in rodents mediated by GLP-1 receptor agonist stimulating calcitonin gene expression [5, 118]. However, humans have shown low GLP-1 receptor expression in thyroid C-cells, which may be an indication of species-specific differences in GLP-1 receptor expression [118].

## 2. Conclusions

With the current diabetes pandemic running in parallel to the one for obesity, there is a growing need to identify pathophysiologic links underlying cancer risk and mortality in this high risk population. The diabetes/metabolic syndrome certainly appears to increase the risk of certain malignancies, including breast, endometrial, colorectal, hepatocellular, and possibly others. Since DM2 may be preventable and certainly treatable with lifestyle modification and pharmacotherapy, cancer risk may also be lowered by these strategies. Certain anti-diabetes therapies (e.g., biguanides) may become the adjuvant chemotherapy of the future. However, large-scale controlled trials are warranted. The role of exogenous insulin administration for cancer risk in diabetes remains controversial; further clinical trials are needed. 

## Figures and Tables

**Table 1a:** (a) Population studies of oral antidiabetic medications and cancer risk

Study	Publish year country (study Yrs)	Increased risk (OR with 95% CI)		Cancer type(s)
Evans et al. [79] case-control hospitalized patients	2005 Scotland (1993–2001)	No (i) Metformin (OR 0.77)		None specified (All incidence of cancers)

Bowker et al. [80] retrospective	2006 Canada (1991–1996)		Yes (i) SFU compared to metformin (HR 1.3, *P* = .012) (ii) Insulin (HR 1.9, *P* = .0001)	None specified (Looked specifically at cancer-related mortality and not cancer type.)

Monami et al. [81] case control	2009 Italy (1998–2004)	After >36 months of use: No (i) Acarbose (OR 0.77) (ii) Glicazide (OR 0.40) (iii) Glitazones (OR 1.05) (iv) Insulin (OR 0.91) (v) Metformin (OR 0.28) (vi) Other SFU (OR 1.05) (vii) Repaglinide (OR 0.87)	After >36 months of use: Yes (i) Glibenclamide (OR 2.62, *P* = .009)	GI breast genital tract (male/female) pancreas lung

Li et al. [64] case control hospitalized patients	2009 United States (2004–2008)	No (i) Metformin (OR 0.38)	Yes (i) Insulin (OR 4.99) (ii) Insulin secretagogues (OR 2.52) (iii) TZD's (OR 1.55)	Pancreas (adenocarcinoma)

Mannucci et al. [82] case control	2010 Italy (1998–2007)	No (i) Other insulin: lispro, aspart, and human insulin (ii) Metformin	Yes (i) ≥0.3 IU/kg/d of glargine	GI hepatic pancreatic lung leukemia/lymphoma breast urogenital prostate 20 “other cancers”

**Table 1b:** (b) Meta-analyses of insulin and cancer

Study	Publish Year country (study Yrs)	Total number of studies	Total (*n*)	Insulin analyzed	Increased risk?	
Dejgaard et al. [83] Meta-analysis of randomized controlled Novo Nordisk trials Type I and Type II	2009 Denmark	21	8693	Detemir NPH glargine	No (i) Glargine and detemir had similar risk	Yes (i) NPH had more risk than detemir

Home et al. [84] Meta-analysis of randomized controlled Sanofi Aventis trials Type I and Type II	2009 United Kingdom	31	10,880	Glargine	No	

**Table 1c:** (c) Population studies of insulin and cancer risk

Study	Publish year country (study Yrs)	Total (*n*)	Insulin analyzed	Increased risk?	
Yang et al. [85] retrospective cohort	2004 United Kingdom (1987–2002)	24,918	Insulin analogues (types not specified)		Yes colorectal cancer
Donadon et al. [86] case control	2008 Italy (1994–2006)	955	Insulin analogues (types not specified)		Yes hepatocellular cancer in insulin-treated males

Hemkens et al. [87] retrospective cohort	2009 German (1998–2005)	127,031	Aspart, glargine, human insulin, lispro		Yes may be dose dependent

Jonasson et al. [88] retrospective cohort	2009 Sweden (2005–2007)	114,841	Glargine	No	

Colhoun et al. [89] retrospective cohort	2009 Scotland (2002–2005)	36,254	Glargine	No	

Currie et al. [90] retrospective cohort	2009 United Kingdom (2000–2005)	62,809	Insulin analogues (types not specified) oral agents	No	

Rosenstock et al. [91] randomized control	2009 United States (5 years)	1017	NPH, glargine	No	

Yang et al. [22] retrospective cohort	2010 Hong Kong (1996–2005)	4623	Insulin analogues (types not specified)	No	

## Abbreviations

DM2: Type 2 diabetes mellitus
BMI: Body mass index
IGF: Insulin-like growth factor
IR: Insulin receptor
Ras: Rat sarcoma protein
PKB: Protein kinase B
mTOR: mammalian target of rapamycin
TNF *α*: Tissue necrosis factor *α*
PI3K: Phosphatidylinositol-3 kinase
GLUT4: Glucose transporter type 4
MAPK: Mitogen-activated protein kinase
IL-6: Interleukin-6
Akt: Serine/threonine-specific protein kinase
MCP-1: Monocyte chemoattractant protein-1
VEGF: Vascular endothelial growth factor
AMP: 5′ adenosine monophosphate activated protein
IRS: Insulin receptor substrate
SFU: Sulfonylurea
TZD: Thiazolidinedione
GLP-1: Glucagon-like peptide-1.
